# In-depth molecular analysis of lymphomas with lymphoplasmacytic differentiation may provide more precise diagnosis and rational treatment allocation

**DOI:** 10.1007/s00277-023-05531-9

**Published:** 2023-11-11

**Authors:** Andrea Brunner, Gudrun Carolina Thalhammer-Thurner, Wolfgang Willenbacher, Margot Haun, Bettina Gudrun Zelger, Ella Willenbacher

**Affiliations:** 1 Department of Pathology, Neuropathology and Molecular Pathology, Innsbruck Medical University, Innsbruck, Austria; 2 Internal Medicine V, Haematology & Oncology, Innsbruck Medical University, Innsbruck, Austria; 3 Syndena GmbH, Connect to Cure, Innsbruck, Austria; 4 Institute of Pathophysiology, Innsbruck Medical University, Innsbruck, Austria

**Keywords:** Lymphoplasmacytic lymphoma, Waldenstroem’s macroglobulinemia, Small B-cell lymphomas with plasmacytic differentiation, Mutations, Next-generation sequencing

## Abstract

We performed a molecular analysis of formalin-fixed paraffin embedded and decalcified bone marrow trephine biopsies of 41 patients with a B-cell disorder with lymphoplasmacytic differentiation to enable a more precise diagnosis and to describe potentially prognostic and therapeutic relevant mutations. Analysis was performed with a commercially available next-generation sequencing (NGS) lymphoma panel (Lymphoma Solution, SophiaGenetics). Results were correlated with clinical and pathological parameters. Our group covered a spectrum of B-cell disorders with plasmacytic differentiation ranging from Waldenstroem’s macroglobulinemia (WM), to small-B-cell lymphomas with plasmacytic differentiation (SBCL-PC) to IgM myeloma (MM). The most helpful diagnostic criteria included morphology and immuno-phenotype as a prerequisite for the interpretation of molecular analysis. *MYD88* mutation was present in nearly all WM, but also in 50% of SBCL-PCs, while MM were consistently negative. Driver mutations, such as *TP53*, were already detectable early in the course of the respective diseases indicating a higher risk of progression, transformation, and reduced progression-free survival. In addition, we report on a novel BIRC3 frameshift mutation in one case of a progressive WM. Our data indicate that patients with LPL/WM might benefit from thorough pathological work-up and detailed molecular analysis in terms of precise diagnosis and targeted treatment allocation.

**Supplementary Information:**

The online version contains supplementary material available at 10.1007/s00277-023-05531-9.

## Introduction

Lymphoplasmacytic lymphoma (LPL) is a B-cell neoplasia defined by a variable mixture of lymphocytes, lymphocytes with plasmacytic differentiation and plasma cells. It is known as Waldenstroem’s macroglobulinemia (WM), when presenting with IgM paraproteinemia and bone marrow involvement [1, 2]. However, (lympho)plasmacytic differentiation can also be found in other low-grade B-cell lymphomas, mainly in marginal zone lymphoma (MZL) or chronic lymphatic leukemia (CLL), but also in mantle cell lymphoma (MCL) [1, 3–6]. Beside morphological characteristics, the immune phenotype may overlap between low-grade lymphomas, resulting in clear diagnostic complexities [3, 4]. In fact, LPL/WM has no distinctive immune phenotype, differentiating it from MZL making a clear diagnosis nearly impossible [3]. This in turn leads to a diagnosis of a small B-cell lymphoma with plasmacytic differentiation (SBCL-PC) [4]. In addition, elevated IgM levels, lymphoid differentiation, and overlapping immune phenotype can also be seen in MM [7–9]. The detection of *MYD88* mutations, most frequently the *MYD88 L265P* mutation, which is found in more than 90% of WM, was believed to reduce this diagnostic dilemma [4, 10]. However, while LPL/WM can be negative for *MYD88* mutations, rare cases of MZL, CLL, and even MCL can harbor a *MYD88* mutations, while only MMs are consistently negative [1, 3, 4]. A variety of clinical studies has dealt with the impact of molecular genetics in LPL/WM [5, 11–16]. The vast majority of WM demonstrates *MYD88* mutations associated in up to 40% of cases with mutations on *CXCR4* [5]. Lack of *MYD88* mutation in LPL/WM, however, predicts worse outcome with higher risk of transformation, therapy-related myelodysplastic syndrome, as well as shorter time to transformation compared to MYD88 mutated patients [5, 11, 12]. Responses in MYD88-wild-type patients seem to be even worse with respect to BTK-inhibitors with the possible exception of zanubrutinib [5, 11, 12, 17]. While *MYD88* is nearly always mutated in *L265P*, up to 40 different *CXCR4* mutations to date occur in LPL/WM [5]. *CXCR4* mutated cases have high serum IgM levels resulting more frequently in hyperviscosity of blood [5, 14]. In addition, the depth of response and progression-free survival partly depends on *CXCR4* mutations when receiving BTK inhibitor-based treatment or Venetoclax, the first representative BCL-2 inhibitor [5, 13–15, 18]. No such impact is observed using upfront treatment with proteasome inhibitors [16]. Similarly, TP53 mutations, though only rarely found in WM are associated with poor outcome, while its role in resistance to BTK-inhibitor therapy is still under investigation [14, 19–22]. Further alterations found in LPL/WM include mutations on *KMT2D*, *ARID1A, CD79A* and *B, NOTCH2, PRDM1*, and *TRAF3*, but their impact on prognosis and treatment remains to be determined [14, 23, 24].

We have previously dealt with the presence of *MYD88 L265P* mutation in LPL/WM [10, 25]. We showed that digital PCR is a sensitive tool to detect this mutation in formalin-fixed paraffin-embedded (FFPE) and decalcified bone marrow trephine biopsies of patients with LPL/WM [25]. In the present study, we attempted to integrate morphology and molecular pathology for a more precise diagnosis by performing next-generation sequencing (NGS) in primary and relapsed LPL/WM. Our results indicate that patients might benefit from a broader molecular analysis already upon diagnosis to allocate the appropriate treatment.

## Material and methods

### Patients

Forty-one patients were identified either from the database of the University Clinics of Internal Medicine V, Hematology and Oncology or from the Institute of Pathology, Neuropathology and Molecular Pathology. The patients have been diagnosed with a neoplastic B-cell disorder presenting with a plasmacytic or lymphoplasmacytic differentiation between 1993 and 2016*.* Bone marrow trephine biopsies were collected and re-evaluated, including follow-up biopsies, if available. Clinical data such as IgM level at diagnosis, presence or absence of osteolysis, cytogenetics and information about disease progression or transformation were assessed from the patient chart.

The study was conducted according to the ICH-GCP guidelines and the declaration of Helsinki. Ethical approval was obtained from the ethical committee of the Medical University of Innsbruck (EK-Nr.1362/2020).

### Re-evaluation and immunohistochemistry

A careful review was performed of all bone marrow biopsies including routinely performed immunohistochemistry to assess morphological parameters such as infiltration pattern, volume of infiltration and number of mast cells and immune-phenotype.

Pattern of infiltration was defined according to Garcia-Reyero J et al. [26] as:

- Paratrabecular and interstitial, either patchy or nodular and/or diffuse
- Only interstitial, either patchy or nodular or diffuse or diffuse-solid

A mast cell count on routinely performed Giemsa stains was done in five representative high-power fields (HPFs) in each biopsy.

Re-evaluation of immunohistochemistry included presence or absence of aberrant marker expression such as CD23 or CD5, positivity for plasma cell markers and evaluation of clonality using either immunohistochemistry or chromogen in situ hybridization (Ventana, Tucson, USA) on an automated immunostainer (Benchmarks Ultra, Ventana, Tucson, USA).

### DNA isolation

DNA from FFPE tissue was extracted using a QIAamp DNA FFPE Tissue Kit (Qiagen, Hilden, Germany). DNA isolation was conducted according to the manufacturer’s instructions. Briefly, 3 × 5 µm slices or 6 × 5 µm slices (depending on the size of the tissue) were prepared in DNAse- and RNAse-free Eppendorf tubes. Paraffin was removed by a Xylol wash. The samples were lysed by use of proteinase K and heat incubated at 90 °C to reposition formalin crosslinking. Then the samples were transferred into QIAamp MinElute columns possessing special DNA binding properties. Residual impurities were washed away by use of the provided buffers. Finally, the respective DNA was eluted from the column membrane. The pure and concentrated DNA samples were stored at − 20 °C until further use.

### Next-generation sequencing

NGS was performed using a commercially available lymphoma panel (Lymphoma Solution, SophiaGenetics, Geneva, Switzerland). This panel includes 54 genes relevant for lymphomagenesis, including those found in lymphomas with plasmacytic differentiation, such as *MYD88, CXCR4, ARID1A, KMT2D*, and those associated with disease progression or transformation such as TP53. Data were evaluated using the platform provided by SOPHIA GENETICS (SOPHIA DDM version 5.10.28.2). A cutoff of 5% was determined for the variant fraction in order to be regarded as a reliable mutation.

### Statistics

For statistical analysis, SPSS 26 software was used. To test for correlations with clinical-pathological parameters the chi-square test was used. To determine the optimal cutoffs for continuous variables (such as number of mast cells) considering diagnostic ability, progression, transformation, and overall survival ROC analysis was performed and AUC was interpreted as follows: 0.5–0.6 = no discrimination, 0.6–0.7 = poor, 0.7 to 0.8 = acceptable, 0.8 to 0.9 = excellent, > 0.9 = outstanding. The optimal cutoffs were calculated using Youden´s index if AUC was > 0.7 [27, 28] (see suppl. Figure 1 and suppl. Table 1). Survival analysis was performed using Kaplan–Meier survival analysis compared with the log rank test. Multivariate analysis was done using Cox regression. A *p* value < 0.05 was considered significant.

## Results

### Patient’s characteristics and morphology

Eight patients had to be excluded because the FFPE material was insufficient for DNA isolation (< 20% neoplastic cells in the bone marrow) or NGS did not provide reliable results due to reduced DNA quality. Thus, finally, our study group included 30 patients diagnosed with LPL, including 24 cases with WM and 6 with SBCL-PC. Three cases of (IgM) MM were included as a proof of principle. Table 1 summarizes the clinical and Table 2 the pathological characteristics of the final study group.

**Table 1:** Clinical characteristics of the 33 patients included in the study (*includes complex karyotypes, T3/3q, T18/18q, del 6q and 14q, del 13q, del17p and loss of Y chromosome; *N* = number of cases)

		WM *N* (%)	SBCL-PC *N* (%)	(IgM) MM *N* (%)	*p* value
Age	< 64.5	16 (66.7%)	1 (6.7%)	2 (66.7%)	0.081
	> 64.5	8 (33.3%)	5 (83.3%)	1 (33.3%)	
Gender	Male	14 (58.3%)	4 (66.7%)	2 (66.7%)	0.909
	Female	10 (41.7%)	2 (33.1%)	1 (33.1%)	
IgM at diagnosis	> 850 mg/dl	9 (37.5%)	1 (16.7%)	0 (0.0%)	0.039
	< 850 mg/dl	2 (8.3%)	3 (50.0%)	0 (0.0%)	
	Unknown	13 (54.2%)	2 (50.0%)	3 (100.0%)	
Progression	Yes	9 (37.5%)	3 (50.0%)	2 (66.7%)	0.365
	No	10 (41.7%)	3 (66.7%)	0 (0.0%)	
	Unknown	5 (20.8%)	0 (0.0%)	1 (33.3%)	
Transformation	Yes	3 (12.5%)	2 (33.3%)	0 (0.0%)	0.487
	No	16 (66.7%)	4 (66.7%)	2 (66.7%)	
	Unknown	5 (20.8%)	0 (0.0%)	1 (33.3%)	
Death	Yes	8 (33.3%)	3 (50.0%)	2 (66.7%)	0.414
	No	11 (48.8%)	3 (50.0%)	0 (0.0%)	
	Unknown	5 (20.8%)	0 (0.0%)	1 (33.3%)	
Osteolysis at diagnosis	Yes	1 (4.2%)	1 (16./%)	0 (0.0%)	0.174
	No	10 (42.7%)	4 (66.7%)	0 (0.0%)	
	Unknown	13 (54.2%)	1 (16.7%)	3 (100.0%)	
Cytogenetics at diagnosis	Pathological*	3 (12.5%)	4 (66.4%)	0 (0.0%)	0.034
	Normal	4 (16.4%)	0 (0.0%)	0 (0.0%)	
	Unknown	17 (70.8%)	2 (33.3%)	3 (100.0%)

**Table 2:** Pathological and molecular characteristics of the 33 patients included in the study (*N* = number of cases)

		WM *N* (%)	SBCL-PC *N* (%)	(IgM) MM *N* (%)	*p* value
Immunophenotype	CD20 + vs38c + CD5-CD23-CyclinD1-	24 (100.0%)	4 (66.7%)	0 (0.0%)	< 0.001
	CD20 + vs38c + **CD5 + CD23 + **CyclinD1-	0 (0.0%)	2 (33.3%)	0 (0.0%)	
	**CD20-/ + vs38c** + CD5-CD23-CyclinD1-/ +	0 (0.0%)	0 (0.0%)	3(100%)	
Volume of infiltration	$\le$ 45%	12 (50.0%)	3 (50.0%)	1 (33.3%)	0.859
	> 45%	12 (50.0%)	3 (50.0%)	2 (66.7%)	
Pattern of infiltration	Paratrabecular/interstitial	18 (75.0%)	3 (50.0%)	0 (0.0%)	0.029
	Interstitial only	6 (25.0%)	3 (50.0%)	3 (100.0%)	
Clonality	Clonal	10 (41.7%)	3 (50.0%)	3 (100.0%)	0.261
	Polyclonal	1 (4.2%)	1 (16.7%)	0 (0.0%)	
	Unknown	13 (54.2%)	2 (33.3%)	0 (0.0%)	
Mast cells	$\le$ 17.50	6 (25.0%)	5 (83.3%)	3 (100.0%)	0.004
	> 17.50	18 (75.0%)	1 (16.6%)	0 (0.0%)	
Number of mutations	≤ 2	16 (66.6%)	1 (16.7%)	3 (100.0%)	0.018
	> 2	8 (33.3%	5 (83.3%)	0 (0.0%)	
*MYD88*	Yes	23 (95.8%)	3 (50.0%)	0 (0.0%)	< 0.001
	No	1 (4.2%)	3 (50.0%)	3 (100.0%)	
*CXCR4*	Yes	6 (25.0%)	0 (0.0%)	0 (0.0%)	0.253
	No	18 (75.0%)	6 (100.0%)	3 (100.0%)	

**Fig. 1:**
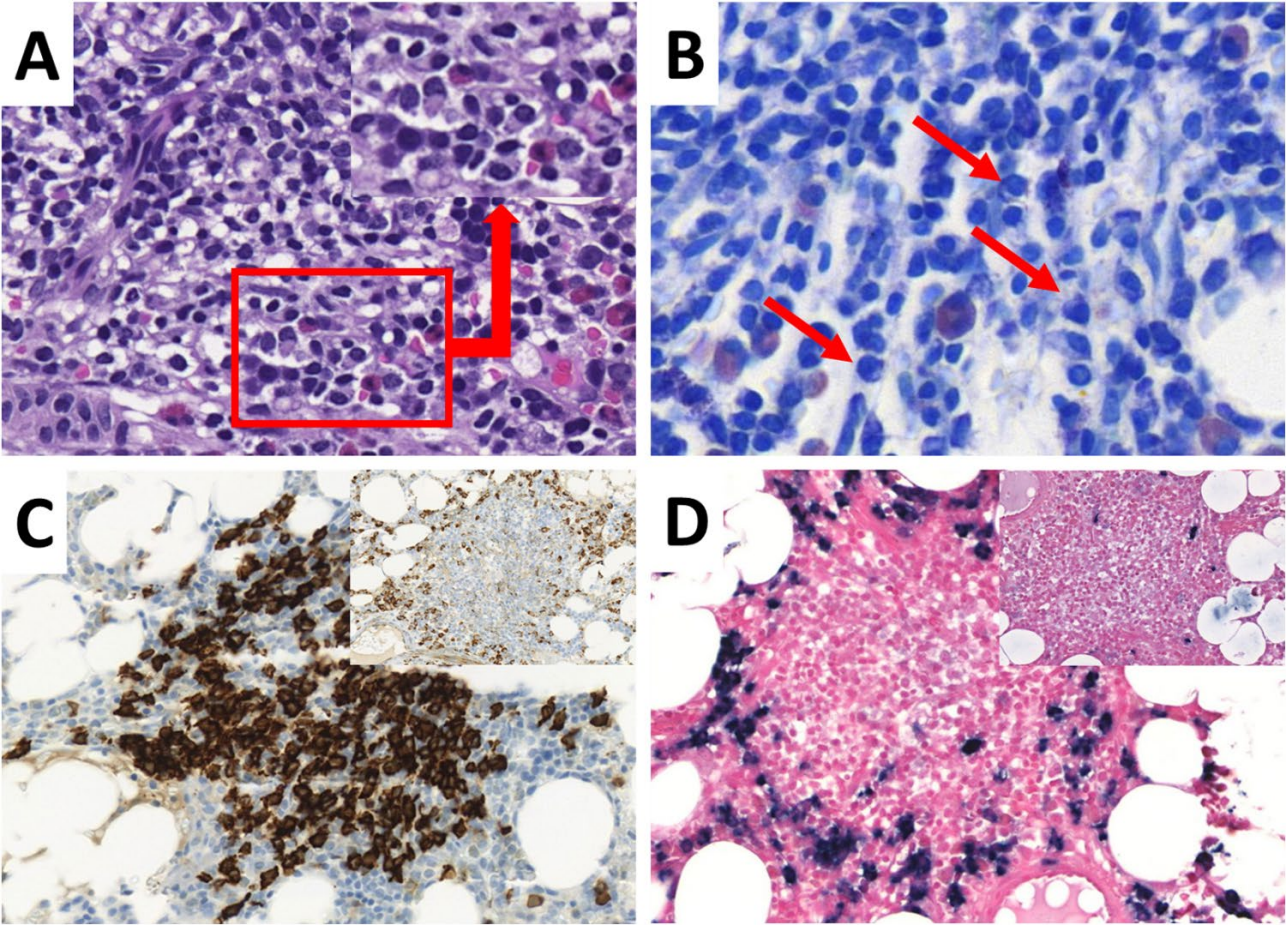
Classical WM with an admixture of lymphocytes, plasmacytoid lymphocytes and plasma cells (**A** H&E; inlet highlights plasma cell differentiation; **B** GIEMSA; plasma cells see arrows); CD20 highlights lymphoma cells within nodular aggregates (**C**), while plasma cells are located at the edge of the infiltrate (see inlet: immunohistochemistry for vs38c). Plasma cells show clonal restriction by chromogen in situ hybridization (INFORM ISH kappa and lambda, Ventana Medical Systems, Tucson, USA) for the light chains Kappa (**D**) and Lambda (see inlet) (**A**, **C**–**D** magnification 20 × ; **B** magnification 40 ×)

Most lymphomas had a classical WM morphology. Some of them had a more plasmacytoid differentiation and Dutcher bodies were prominent. Immune phenotype was also typical for WM with negativity for CD5 as well as CD23 and variable expression of plasma cell markers (see Fig. 1A–D) in most of the cases.

**Fig. 2:**
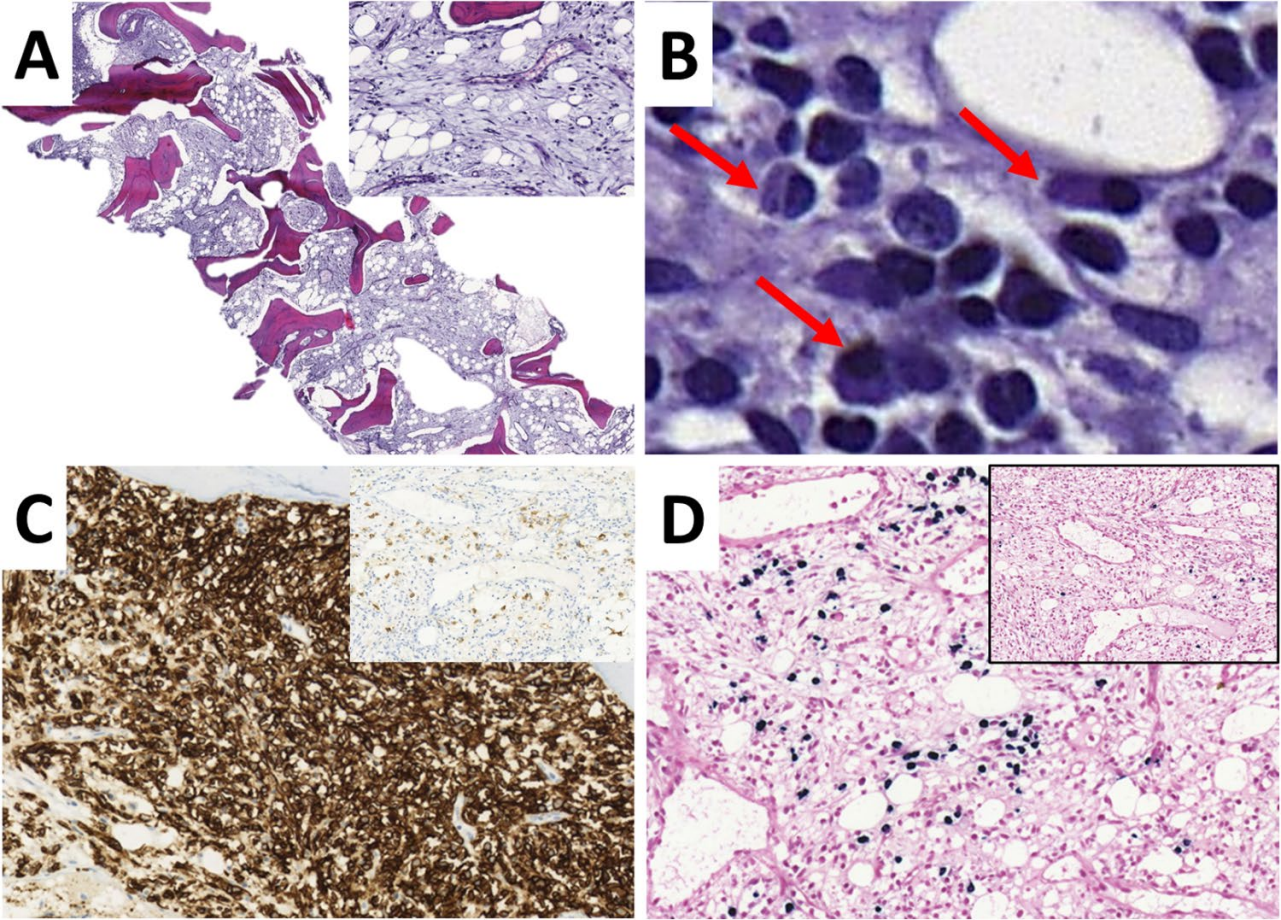
WM wild-type with marked osteosclerosis and fibrosis at diagnosis (**A** fibrosis is highlighted in the inlet; H&E). The tumor was composed of a mixture of lymphocytes, lymphoplasmacytoid cells, and plasma cells (**B**; arrows show plasmacytoid cells and plasma cells; H&E). Tumor cells were CD20 positive and partially expressed plasma cell markers (**C** immunohistochemistry for CD20; inlet: CD138) Plasma cells were clonal (in situ hybridization for light chains kappa and lambda (**D** Kappa; inlet = Lambda). A DLBCL was diagnosed synchronously in lung and CNS

The only wild-type WM showed progressive fibrosis and osteosclerosis and had a synchronous diagnosis of diffuse large B-cell-lymphoma (DLBCL) in lung and central nervous system (CNS) (see Fig. 2A–D).

**Fig. 3:**
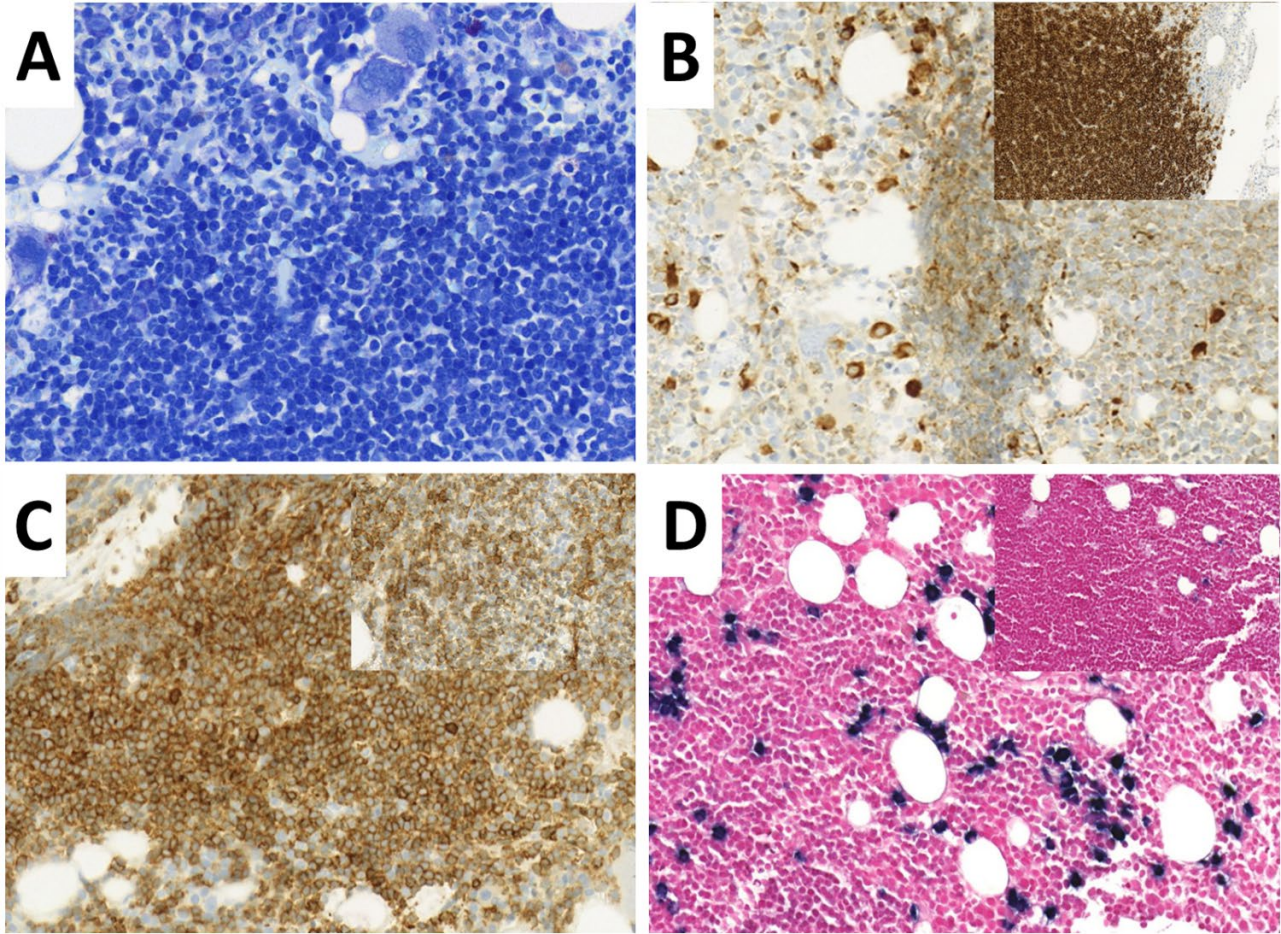
SBCL-PC finally classified as CLL with features reminiscent of LPL/WM including plasmacytic differentiation with paratrabecular and interstitial infiltrate of lymphocytes (**A** GIEMSA) admixed with plasma cells (**B** immunohistochemistry for vs38c), while the majority of the neoplastic cells is CD20 positive (see inlet in **B**). Additionally, neoplastic cells express CD5 and CD23 (**C** immunohistochemistry for CD5 and inlet in **C** immunohistochemistry for CD23). Chromogen in situ hybridization for light chains kappa and lambda showed a clear predominance of kappa positive light chains (**D** kappa and inlet in **D** lambda). (all magnification 20 ×)

Two of the six SBCL-PC showed a more variable immune phenotype with additional expression of CD5 and CD23, but also weak or focal expression of plasma cell markers. While one case had polyclonal plasma cells in the background and did not show a MYD88 mutation, the other case was positive for a MYD88 mutation and had monoclonal plasma cells. Both were finally classified as CLL (see suppl. Table 2). Nevertheless, the diagnosis of MYD88 mutations together with clonal plasma cells suggests the existence of a spectrum between B-CLL and classical WM (see Fig. 3A–D).

The other four SBCL-PCs had a more classical WM morphology with paratrabecular and interstitial nodular infiltration (two cases), a striking plasmacytic differentiation (one case) or an interstitial pattern of infiltration being suggestive of a MZL (one case). MMs showed clonal plasma cell proliferations with CD20 and cyclin D1 expression in two of three cases. The best distinguishing features as highlighted in Table 1 for the diagnosis of LPL/WM were morphological parameters, such as infiltration pattern (*p* = 0.029) and mast cell count (*p* = 0.001), immune phenotype (*p* < 0.001) and presence of *MYD88* mutation (*p* < 0.001) (see Table 1).

### Next-generation sequencing and its impact on diagnosis

Overall mean number of mutations detected per case was 2 (range 0–12). MYD88 mutations were found in roughly 95% of the cases with WM with CXCR4 mutations being present in 25%. The majority of MYD88 mutations were MYD88 L265P mutations, but in two WM cases, different missense mutations were detected in exon 3 (*MYD88 L229S*) (see Fig. 1) and exon 1 (*MYD88 P34S*). Other mutations in WM included mutations of *ARID1A* (20.8%), *KMT2D* (12.5%), *TP53* (8%), *POT1* (8%), *TNFAIP3* (8%), and *BIRC3* (4%). One case of WM was wild-type with synchronous DLBCL and mutations on *CREBBP, EZH2, CARD11, PIM1, SOCS1, STAT6,* and *TNFRSF14. MYD88 L265P* was detected in 50% of SBCL-PC with none of them harboring a CXCR4 mutation or *ARID1A* mutation. Other mutations in this group included *POT1* (4%), *MEF2B* (4%), *FBXW7* (4%), and *XPO1* (4%). Taking into account morphology, molecular analysis and clinical characteristics a final diagnosis was achieved in four of six SBCL-PC (2 CLL, one MZL, one WM), while two lymphomas remained unclassifiable (see suppl. Table 2). (IgM) MM cases lacked *MYD88* mutation and any other relevant of the analyzed mutations except *TP53* in two of three cases (66%). Thus, our results are overall in line with the current literature [1, 10, 14, 21, 23].

Table 3 highlights the type of mutations present in our cases.

**Table 3:** Most frequent mutations detected in the study group including primary diagnoses as well as biopsies at relapse (*N* = number of biopsies (total 43) evaluated)

Type of mutation	Missense mutation (%)	Nonsense mutation (%)	Frameshift mutation^2^ (%)	Splice-site mutation (%)
*MYD88*	37 (86.04%)^1^	-	-	-
*CXCR4*	-	2 (4.6%)	10 (23.2%)	-
*ARIDA1*	1 (2.3%)	1 (2.3%)	6 (13.9%)	1 (2.3%)^3^
*KMT2D*	5 (11.6%)	-	1 (2.3%)	-
*TP53*	4 (9.3%)	-	-	4 (9.3%)^3^
*POT1*	2 (4.6%)	-	1 (2.3%)	-
*TNFAIP3*	3 (6.9%)	-	-	-

^1^Includes two mutations other than MYD88 L265P

^2^Includes frameshift insertions in ARID1A and POT1 and a frameshift deletion in KMT2D

^3^Substitution—intronic in two patients, one with repeated biopsies

To compare molecular analysis with other clinical parameters, we carefully reviewed the literature on known driver mutations in LPL/WM and CLL and we defined molecular groups as follows:

- *Group 1:* All patients with *MYD88* and/or *CXCR4* and/or *ARIDA1* mutation without other additional mutations, which are known as driver mutations in lymphomas.
- *Group 2:* All patients with *MYD88* and/or *CXCR4* and/or *ARIDA1* mutations plus additional mutations, which are known driver genes in lymphomas (*TP53, TNFAIP3, BIRC3, FBXW7, POT1 XPO1*).

The three cases of IgM MM were not grouped in either of the categories and thus were not included in further statistics.

When applying the cutoff for mast cell counts as determined by ROC in molecular group 1 significantly more often a mast cell count above the cutoff was observed than in molecular group 2 (17/21 (81%) above the cutoff versus 2/9 (22%) above the cutoff; *p* = 0.002). The same was observed for immune phenotype (21/21 (100%) versus 7/9 (78%); *p* = 0.025). However, these results most likely merely depict the fact that group 2 included more cases which were difficult to interpret diagnostically reflected by the immunophenotype and mast cell count.

In seven patients, follow-up biopsies were available for analysis, resulting in 43 finally analyzed bone marrow biopsies. Five of them had progressive disease and *MYD88* mutation was the most frequently observed mutation except for one case which had a BIRC3 frameshift mutation in all examined specimens, so far not described in the literature (see Table 5). In four patients with subsequent DLBCL, additional oncogenic mutations were detected in the WM component before the appearance of the aggressive lymphoma component (see Table 4).

**Table 4:** Molecular characteristics of the seven patients in the study, where more than one biopsy was available for NGS

	1	2	3	4	5	6	7
Age	62	59	67	54	52	43	72
Gender	Male	Female	Female	Male	Male	Male	Female
Diagnosis	MW	MW	MW	MW	MW	MW	SBCL-PC
Diagnosis at transformation	-	-	DLBCL, GCB with involvement of lung and CNS	DLBCL, CD5 + , most likely non-GCB	DLBCL, Burkitt-like	-	DLBCL, non-GCB in CNS; primary CNS lymphoma
Progression	Yes	Yes	No	Yes	No	Yes	Yes
Biopsy 1	**02/2006**	**05/2006**	**11/2007**	**12/2007**	**12/2007**	**07/2010**	**02/2010**
	MYD88 L265P CXCR4 H341Wfs*8, ARIDA1 G2087R	MYD88 L265P ARIDA1 P225Afs*175	CREBBP A1435Pfs*77, EZH2 Y641F, PIM1 Y129*, CARD 11 N357V, SOCS1 S71R, F58L, F135L A132V and F148S, STAT6 D419V, TNFRSF14 C99G	MYD88 L265P, CXCR4 H341Gfs*7 ***TP53 unknown, KMT2D R4825G***	MYD88 L265P, CXCR4 T346Rfs*3 ***TP53 C176Y, TNFAIP3, R136C* + *T647P***	MYD88 L265P, ARID1A P225Afs*175, BIRC3 A434Gfs*12	*MYD88 L265P, *KMT2D G152Rfs*56
Biopsy 2	**06/2007**	**01/2007**	**11/2007**	**07/2009**	**01/2009**	**03/2011**	**03/2010**
	MYD88 L265P, CXCR4 H341Wfs*8	*MYD88 L265P*	Performed within one month, no further NGS possible	MYD88 L265P, CXCR4 H341Gfs*7, ***TP53 unknown, KMT2D R4825G***	MYD88 L265P, CXCR4 T346Afs*3, ***TP53 C176Y****, ****TNFAIP3 R136C* + *T647P***	MYD88 L265P, ARID1A P225Afs*175, BIRC3 R434Gfs*12	Performed within one month, no further NGS possible
Biopsy 3			04/08	**11/2009**	**10/2015**	**01/2013**	
	-	-	Only necrosis, no further NGS, DLBCL in lung	MYD88 L265P, CXCR4 H341Gfs*7, ***TP53 unknown, KMT2D R4825G***	Minimally infiltrated by WM; no further NGS	MYD88 L265P, ARID1A P225Afs*175, BIRC3 R434Gfs*12	-
Biopsy 4				**01/2011**		**10/2013**	
	-	-	-	MYD88 L265P, CXCR4 H341Gfs*7, ***TP53 unknown, KMT2D R4825G, EP300 T1491S***	-	MYD88 L265P, ARID1A P225Afs*175, BIRC3 R434Gfs*12	-
Biopsy 5						**12/2013**	
	-	-	-	-	-	MYD88 L265P, ARID1A P225Afs*175, BIRC3 R434Gfs*12	-

### Association with disease progression and survival

For analysis of progression-free survival (PFS) and overall survival (OS), we excluded the three MM cases, since these are clinically different and more aggressive entities. In 25 of the remaining 30 patients, information on follow-up concerning disease progression and/or transformation was available.

Table 5 depicts the association of clinical and pathological characteristics with the development of progressive disease, transformation, and death.

**Table 5:** Association of pathological and molecular characteristics with development of progression, disease transformation, and death

		Progression		Transformation		Death	
		Yes	No	Yes	No	Yes	No
Age	< 64.5	6 (24.0%)	2 (8.0%)	3 (12.0%)	5 (20.0%)	4 (16.0%)	4 (16.0%)
	> 64.5	7 (28.0%)	10 (40.0%)	2 (8.0%)	15 (60.0%)	7 (28.0%)	10 (40.0%)
	***p* value**	0.114		0.133		0.687	
Gender	Male	7 (28.0%)	9 (37.0%)	3 (12.0%)	13 (52.0%)	7 (28.0%)	9 (36.0%)
	Female	6 (24.0%)	3 (12.0%)	2 (8.0%)	7 (28%)	4 (16.0%)	5 (20.0%)
	***p* value**	0.271		0.835		0.973	
Mol. group	Group 1	6 (24.0%)	11 (44.0%)	1 (4.0%)	16 (64.0%)	5 (20.0%)	12 (48.0%)
	Group 2	7 (28.0%)	1 (4%)	4 (16.0%)	4 (16.0%)	6 (28.0%)	2 (8.0%)
	***p* value**	***0.015***		***0.01***		***0.032***	
Pattern	Paratrabecular and interstitial	8 (32.0%)	10 (40.0%)	3 (12.0%)	15 (60.0%)	7 (28.0%)	11 (44.0%)
	Interstitial only	5 (20.0%)	2 (8.0%)	2 (8.0%)	5 (20%)	4 (16.0%)	3 (12.0%)
	***p* value**	0.225		0.504		0.409	
Mast cell count	$\le$ 17.5	6 (24.0%)	3 (12.0%)	4 (16.0%)	5 (20.0%)	5 (20.0%)	4 (26.0%)
	> 17.5	7 (28.0%)	9 (36.0%)	1 (4.0%)	15 (60.0%)	6 (24.0%)	10 (40.0%)
	***p* value**	0.271		***0.022***		0.383	

**Fig. 4:**
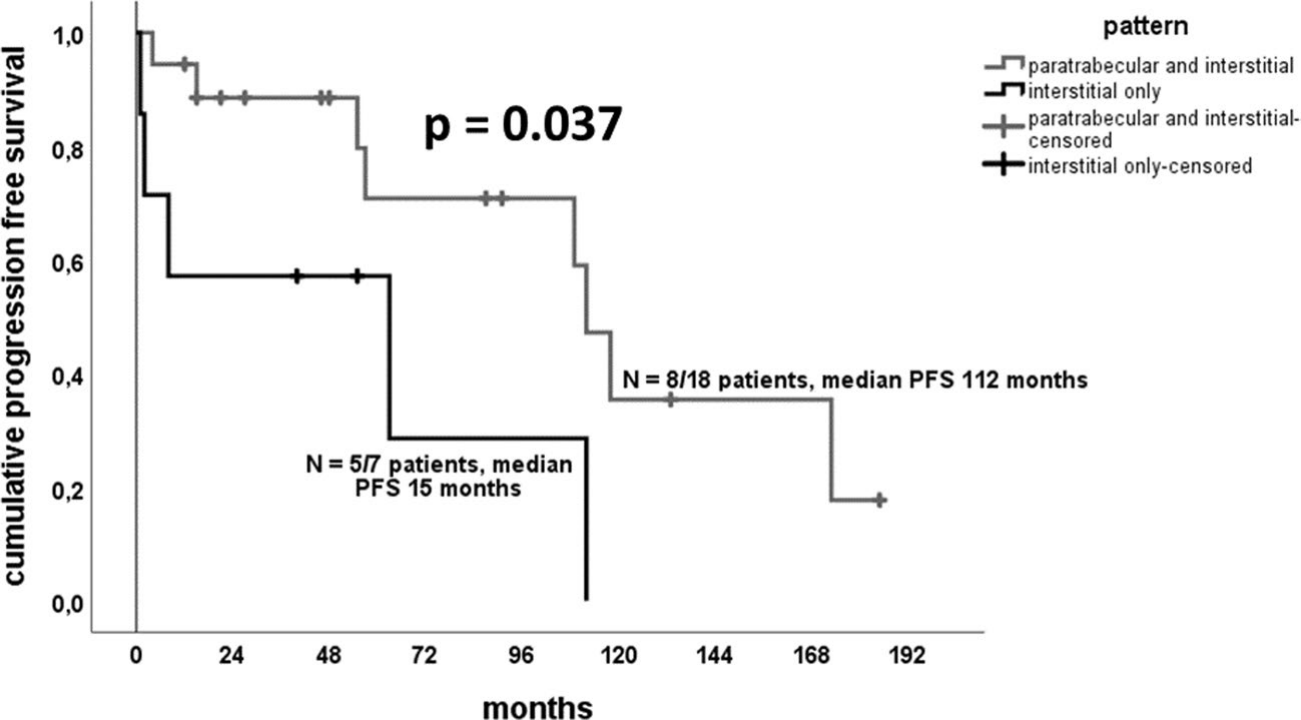
Kaplan–Meier survival analysis for progression-free survival in the 25 patients with WM and SCL-PC: a significantly longer PFS was seen in patients with a paratrabecular and interstitial infiltration pattern compared to those with a purely interstitial pattern (*N* = number of events/number of patients)

Using Kaplan–Meier survival analysis, a significantly better PFS was observed in patients with a paratrabecular and interstitial infiltration pattern compared to those with only interstitial infiltration pattern (8/18 patients, median PFS 112 months versus 5/7patients, median PFS 63 months; *p* = 0.037, see Fig. 4). None of the other parameters, such as molecular group, age or gender significantly influenced PFS, though a tendency was observed that additional driver mutations lead to a worse PFS (6/17 with progressive disease, median PFS 117.00 months versus 7/8 with progressive disease, median PFS 60.62 months; *p* = 0.052).

None of the evaluated parameters significantly influenced OS, though interstitial infiltration pattern showed a slight tendency for worse OS (7/18, median OS 140.90 months versus 4/7, median OS 85.67 months; *p* = 0.097).

## Discussion

In our study, we showed that assessment of classical morphological features in the bone marrow, including infiltration pattern and increased numbers of mast cells still are a prerequisite in the diagnosis of WM [26]. In fact, most WMs had higher mast cell counts and infiltration pattern was often (nodular) paratrabecular and interstitial, less likely only interstitial, irrespective if nodular or diffuse–solid [26, 29]. Though presence of *MYD88 L265P* mutation is helpful in the diagnosis, this mutation is not unique to LPL/WM [1, 30]. Thus, combining careful morphological evaluation with immune phenotyping and molecular analysis in debatable cases is the best way to achieve a proper diagnosis [26, 29]. As already reported in various studies, *MYD88 L265P* was the most frequently found mutation in LPL/WM also in our study followed by mutations on *CXCR4* [1, 15, 23]. In some cases, additional mutations turned up, which are infrequent in LPL/WM. This includes *TP53* mutations associated with *MYD88* and *CXCR4*, which were present in 4% (2 cases) of LPL/WM, a finding that is well in line with the literature [19, 31]. Both patients experienced disease progression and finally developed DLBCL with *TP53* mutation preceding transformation for 4 and 8 years, respectively (see Table 4). In fact, *TP53* mutations predict a worse outcome and rapidly progressive disease [19, 21, 31, 32]. Other mutations include *KMT2D* missense and frameshift mutations, which appear in a variety of lymphomas. *KMT2D* are frequently seen in LPLs and mutations have a possible connection to hypogammaglobinemia, autoimmune phenomena and the Kabucki syndrome, but its role in pathogenesis of LPL is still largely unknown [24, 26, 33]. In two cases, *TNFAIP3* missense mutations were found, which are associated with a diagnosis of B-cell lymphoma including DLBCL [34, 35]. Functionally *TNFAIP3* is a negative regulator in the NFκB pathway and believed to be a tumor suppressor gene [36, 37]. In fact, Compagno et al. [34] who closely investigated DLBCL for the presence and type of *TNFAIP3* mutations reported inactivation by a two-hit mechanism such as a combination of inactivating mutations and/or deletions, which is one possible mechanism that promotes lymphomagenesis. Interestingly loss of chromosome 6q, where *TNFAIP3* gene is located, is not only a frequent event in DLBCL but is also the main cytogenetic event in WM [32, 34, 37]. Indeed, in one of our two patients with a *TNFAIP3* mutation cytogenetics revealed a 6q deletion indicating a similar mechanism. In addition, a *TP53* mutation was present and in one patient, who later on developed transformation. Thus, one might speculate that these findings already indicate a more aggressive disease despite morphologic characteristics of WM. Beside one patient diagnosed as LPL/WM was quite interesting due to morphology, molecular genetics, and clinical outcome. Morphologically, a diffuse lymphoplasmacytic infiltrate in the bone marrow with increased mast cells was seen in the bone marrow and immune phenotype was typically for WM. Molecular analysis revealed a *MYD88* and a *CXCR4* mutation and in addition a frameshift mutation on BIRC3 R434Gfs*12, which was also present in all follow-up biopsies available. Overall tumor burden in the bone marrow remained high (> 40–50%) during the course of disease despite treatment as was allelic burden of *BIRC3* and the patient finally succumbed to the disease. *BIRC3* mutations appear in up to 4% of newly diagnosed CLL, and rarely in other low-grade lymphomas such as MZL and WM [38]. *BIRC3* mutations are mostly nonsense or in-frame variants leading to a loss of function [38, 39]. In brief, degradation of *MAPK3K14*, which is a major driver of the non-canonical NFκB pathway is induced by *BIRC3*, thus loss of *BIRC3* function constitutively activates the non-canonical pathway resulting in cell proliferation and survival [39]. In CLL, *BIRC3* mutations probably indicate a more aggressive course especially in patients treated with fludarabine, cyclophosphamide and rituximab (FCR) [40]. Our case further adds evidence that mutations in *BIRC3* might influence treatment response and outcome. Our group included only one *MYD88* wild-type LPL/WM, which, in accordance with the literature, had a different molecular profile than those with *MYD88* mutations [33]. Furthermore, morphology in the bone marrow showed osteosclerosis and prominent fibrosis features most likely associated with an ongoing transformation to DLBCL, which synchronously occurred in the lung and central nervous system.

In the SBCL-PC group, in addition *POT1, FBXW7, XPO1*, and *MEF2*B had mutations. *POT1, FBXW7,* and *XPO1* mutations belong to subgroups of CLL and are known driver mutations [41–44] but did not occur LPL, so far. However, shared mutations further support the fact that an alliance might exist between these diseases and that mutations are neither specific nor defining for diagnosis*. MEF2B* mutations present in subgroups of DLBCL and follicular lymphoma most likely act as an oncogene, but LPL or CLL did not show these mutations so far [45, 46]. Functionally, *MEF2B* is a transcriptional activator leading to enhanced transcription of *BCL6* and thus promoting lymphomagenesis [45, 46].

Our study has some limitations. First, the number of cases is small since LPL/WM is a relatively rare disease. Second, the number of progressive and transformed cases is even smaller, since these are even rarer events. Boiza-Sanchez et al. [47] lately reported on the molecular analysis of eight cases of WM with development into a DLBCL, which revealed shared mutations including *MYD88 L265P* mutation in the majority of cases. We could only analyze the DLBCL component in one patient with bone marrow infiltration, while in all other patients, high-grade lymphoma occurred extra-medullary and FFPE material was not available in sufficient amounts for further analysis. In this patient, the DLBCL component shared mutations with the preceding LPL/WM with an additional *EP300* mutation (see Table 4).

To sum up, assessment of classical morphological and immunohistochemical features of WM in the bone marrow is still a prerequisite in the diagnosis of lymphomas with plasmacytic differentiation. The presence of driver mutations in LPL/WM hints at a higher risk of progressive disease and transformation and a shorter PFS, further supporting the usefulness of an in-depth molecular analysis already upon diagnosis or at least in progressive disease to allocate the appropriate treatment. For reasons of cost-efficiency in an increasingly troubled healthcare system stratifying patients for further molecular analysis could be done using conventional parameters, including for example atypical immune phenotype, diffuse infiltration pattern, and reduced mast cell component, and should at least be done in treatment-resistant cases.

### Supplementary Information

Below is the link to the electronic supplementary material.

Supplementary file1 (DOCX 76 KB)

## Data Availability

The data that support the findings of this study are available from the corresponding author, [A.B.], upon reasonable request.

## Abbreviations

LPL: Lymphoplasmacytic lymphoma
NGS: Next-generation sequencing
WM: Waldenstroem’s macroglobulinemia
SBCL-PC: Small B-cell lymphoma with plasmacytic differentiation
MM: Multiple myeloma
MZL: Marginal zone lymphoma
CLL: Chronic lymphocytic leukemia
MCL: Mantle cell lymphoma
BTK: Bruton’s tyrosine kinase
FFPE: Formalin-fixed paraffin-embedded
HPF: High-power field
OS: Overall survival
PFS: Progression-free survival
