# Supplementary material for: In-depth molecular analysis of lymphomas with lymphoplasmacytic differentiation may provide more precise diagnosis and rational treatment allocation
Source: Ann Hematol. 2023 Nov 11;103(2):553–63. doi: 10.1007/s00277-023-05531-9 (PMC10798918; doi:10.1007/s00277-023-05531-9)
Supplement: Supplementary file 1 — Supplementary file1 (DOCX 76 KB) [file 277_2023_5531_MOESM1_ESM.docx]

**Supplements**

**
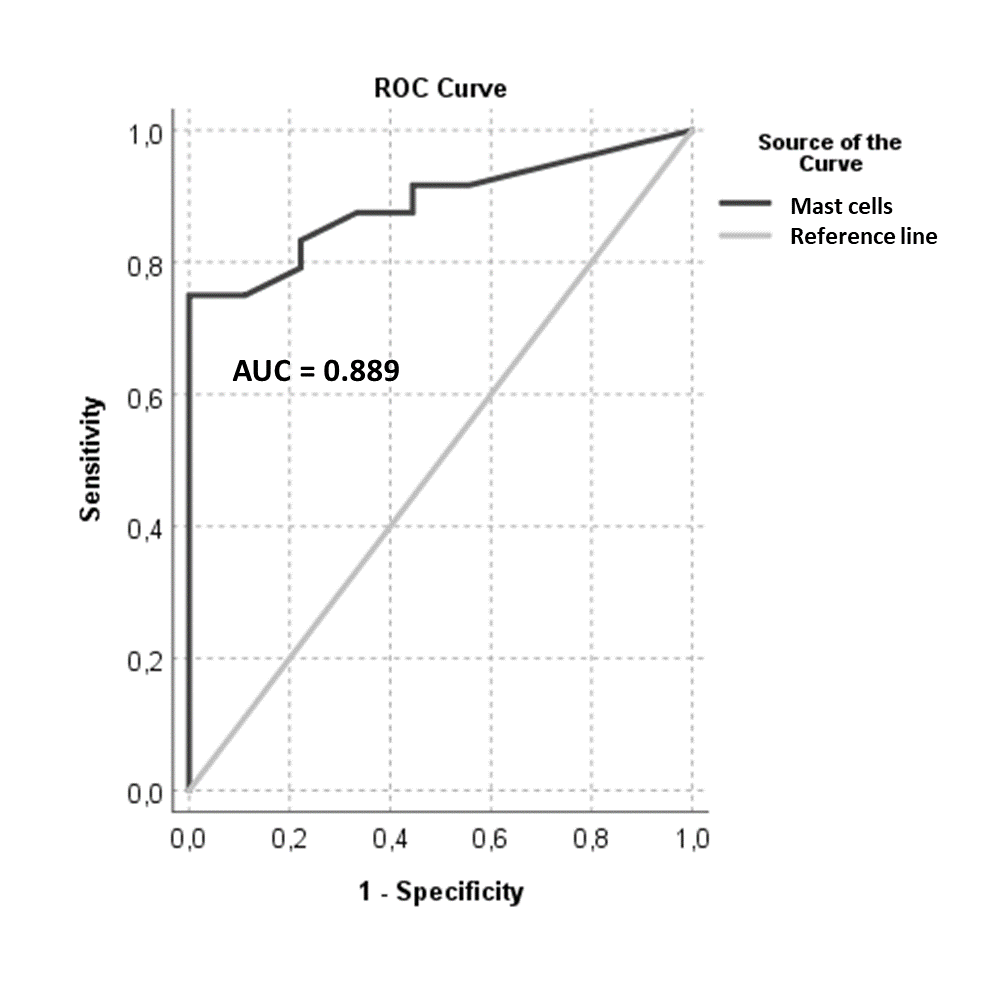
**

**Suppl. fig 1** Exemplary ROC curve for ROC analysis for number of mast cells by diagnosis.

| **Variable** | **State** | **AUC** | **Sensitivity** | **Specificity** | **Cut off** |
| --- | --- | --- | --- | --- | --- |
| **IgM at diagnosis** | **Diagnosis** | ***0.830*** | ***82%*** | ***75%*** | 850.50 mg/dl |
|  | **Progression** | 0.583 | - | - | **-** |
|  | **Transformation** | ***0.727*** | ***75%*** | ***82%*** | 850.50 mg/dl |
|  | **Death** | 0.620 | - | - | **-** |
| **Age** | **Diagnosis** | 0.618 | - | - | - |
|  | **Progression** | ***0.714*** | ***73%*** | ***50%*** | 64.5 years |
|  | **Transformation** | 0.695 | - | - | - |
|  | **Death** | 0.604 | - | - | - |
| **Volume of infiltration** | **Diagnosis** | 0.583 | - | - | - |
|  | **Progression** | 0.636 | 73% | 67% | 45% |
|  | **Transformation** | 0.600 | - | - | - |
|  | **Death** | 0.527 | - | - | - |
| **Mast cells** | **Diagnosis** | ***0.889*** | ***75%*** | ***89%*** | 17.50 |
|  | **Progression** | 0.625 | - | - | - |
|  | **Transformation** | 0.686 | - | - | - |
|  | **Death** | 0.684 | - | - | - |

**Suppl. table 1** Results of ROC analysis with AUC; cut offs were calculated by Youden´s Index, when AUC indicated an acceptable (AUC > 0.7) to excellent (AUC > 0.8) discrimination. In these cases cut offs including sensitivity and specificity are given.

Taking into account morphology, molecular analysis and clinical pathological characteristics a final diagnosis was achieved for nearly all SBCL-PC.

| **Case number** | **1** | **2** | **3** | **4** | **5** | **6** |
| --- | --- | --- | --- | --- | --- | --- |
| **Age at diagnosis** | 72 | 65 | 81 | 74 | 80 | 40 |
| **Gender** | female | male | female | male | male | male |
| **Initial diagnosis** | CLL/WM/  MZL | CLL/WM | CLL/WM | lymphoma/MM | CLL/MW | CLL/WM |
| **Pattern** | interstitial | para-trabecular/  interstitial | interstitial | para-trabecular/ interstitial | para-trabecular/  interstitial | diffuse |
| **Phenotype** | CD20+vs38c+CD23-CD5- | CD20+vs38c+  CD23-CD5- | CD20+vs38c+  CD23-CD5- | CD20+vs38c+  CD23+CD5+ | CD20+vs38c+  CD23+CD5+ | CD20+vs38c+  CD23+CD5+ |
| **Clonality** | NA | NA | yes | yes | yes | no |
| **MYD88 mutation** | yes | yes | no | no | yes | no |
| **CXCR4 mutation** | no | no | no | no | no | no |
| **Other mutations** | KMT2D | none | FBXW7 | no | MEF2B  POT1 | XPO1 |
| **Cytogenetics** | unknown | del13q | unknown | loss of Y | del13q | del17p |
| **Final diagnosis** | MZL | WM | unclassified | unclassified | CLL | CLL |
| **Progression** | yes | no | no | yes | no | yes |
| **Transformation** | yes - DLBCL | no | no | no | no | yes – PCL* |

**Suppl. table 2** Clinical and pathological characteristics of the six patients with SBCL-PC included in the study. Molecular analysis covered a range from LPL to MZL and CLL, with some lymphomas showing overlapping features (*plasma cell leukemia).
